# ^89^Zr-Trastuzumab PET/CT Imaging of HER2-Positive Breast Cancer for Predicting Pathological Complete Response after Neoadjuvant Systemic Therapy: A Feasibility Study

**DOI:** 10.3390/cancers15204980

**Published:** 2023-10-13

**Authors:** D. G. J. Linders, M. M. Deken, M. A. van Dam, M. N. J. M. Wasser, E. M. C. Voormolen, J. R. Kroep, G. A. M. S. van Dongen, D. Vugts, H. M. Oosterkamp, M. E. Straver, C. J. H. van de Velde, D. Cohen, P. Dibbets-Schneider, F. H. P. van Velden, L. M. Pereira Arias-Bouda, A. L. Vahrmeijer, G. J. Liefers, L. F. de Geus-Oei, D. E. Hilling

**Affiliations:** 1 Department of Surgery, Leiden University Medical Center, 2333 ZA Leiden, The Netherlandsd.e.hilling@lumc.nl (D.E.H.); 2 Department of Radiology, Leiden University Medical Center, 2333 ZA Leiden, The Netherlands; 3 Department of Medical Oncology, Leiden University Medical Center, 2333 ZA Leiden, The Netherlands; 4 Department of Radiology and Nuclear Medicine, Amsterdam University Medical Center, 1081 HV Amsterdam, The Netherlands; 5 Department of Internal Medicine, Haaglanden Medical Center, 2512 VA The Hague, The Netherlands; 6 Department of Surgery, Haaglanden Medical Center, 2512 VA The Hague, The Netherlands; 7 Department of Pathology, Leiden University Medical Center, 2333 ZA Leiden, The Netherlands; 8 Department of Radiology, Section of Nuclear Medicine, Leiden University Medical Center, 2333 ZA Leiden, The Netherlands; 9 Department of Nuclear Medicine, Alrijne Hospital, 2353 GA Leiderdorp, The Netherlands; 10 Biomedical Photonic Imaging Group, University of Twente, 7522 NB Enschede, The Netherlands; 11 Department of Radiation Science and Technology, Delft University of Technology, 2628 CD Delft, The Netherlands; 12 Department of Surgical Oncology and Gastrointestinal Surgery, Erasmus MC Cancer Institute, University Medical Center Rotterdam, 3015 GD Rotterdam, The Netherlands

**Keywords:** breast cancer, human epidermal growth factor receptor 2, neoadjuvant therapy, treatment response evaluation, HER2-targeted PET/CT, surgery-free treatment strategy

## Abstract

**Simple Summary:**

In breast cancer patients in whom tumor cells overexpress the protein human epidermal growth factor receptor 2 (HER2), HER2-targeted therapy is the mainstay of neoadjuvant therapy (NAT). Two thirds of these patients respond so well to HER2-targeted therapy that during microscopic analysis of the surgically resected tissue, it becomes apparent there are no vital tumor cells left, classified as complete responders. These patients might not have needed surgery. However, with current imaging techniques such as MRI, it remains difficult to preoperatively assess whether there is residual tumor after NAT or not, so all patients still undergo surgery. This study investigated if a HER2-targeted PET/CT scan can reliably assess the response to NAT. In six patients, a HER2-targeted PET/CT scan was acquired before and after NAT. Two out of six patients had residual tumor at microscopic analysis. Visual assessment of the PET/CT scans correctly predicted the response in 66.7% of cases. When the PET/CT signal in both the scan before and after NAT was quantified and (percentual) changes were calculated, there was a difference in the change of signal between patients with and without residual tumor. This difference, although not statistically significant due to the limited patient number in this study, suggests that quantitative assessment of HER2-targeted PET/CT can be used for accurate response evaluation after NAT.

**Abstract:**

Background: Approximately 20% of invasive ductal breast malignancies are human epidermal growth factor receptor 2 (HER2)-positive. These patients receive neoadjuvant systemic therapy (NAT) including HER2-targeting therapies. Up to 65% of patients achieve a pathological complete response (pCR). These patients might not have needed surgery. However, accurate preoperative identification of a pCR remains challenging. A radiologic complete response (rCR) on MRI corresponds to a pCR in only 73% of patients. The current feasibility study investigates if HER2-targeted PET/CT-imaging using Zirconium-89 (^89^Zr)-radiolabeled trastuzumab can be used for more accurate NAT response evaluation. Methods: HER2-positive breast cancer patients scheduled to undergo NAT and subsequent surgery received a ^89^Zr-trastuzumab PET/CT both before (PET/CT-1) and after (PET/CT-2) NAT. Qualitative and quantitative response evaluation was performed. Results: Six patients were enrolled. All primary tumors could be identified on PET/CT-1. Four patients had a pCR and two a pathological partial response (pPR) in the primary tumor. Qualitative assessment of PET/CT resulted in an accuracy of 66.7%, compared to 83.3% of the standard-of-care MRI. Quantitative assessment showed a difference between the SUV_R_ on PET/CT-1 and PET/CT-2 (ΔSUV_R_) in patients with a pPR and pCR of −48% and −90% (*p* = 0.133), respectively. The difference in tumor-to-blood ratio on PET/CT-1 and PET/CT-2 (ΔTBR) in patients with pPR and pCR was −79% and −94% (*p* = 0.133), respectively. Three patients had metastatic lymph nodes at diagnosis that were all identified on PET/CT-1. All three patients achieved a nodal pCR. Qualitative assessment of the lymph nodes with PET/CT resulted in an accuracy of 66.7%, compared to 50% of the MRI. Conclusions: NAT response evaluation using ^89^Zr-trastuzumab PET/CT is feasible. In the current study, qualitative assessment of the PET/CT images is not superior to standard-of-care MRI. Our results suggest that quantitative assessment of ^89^Zr-trastuzumab PET/CT has potential for a more accurate response evaluation of the primary tumor after NAT in HER2-positive breast cancer.

## 1. Introduction

To date, breast cancer remains the most commonly diagnosed cancer and the leading cause of cancer-related mortality in women, representing about 25% of all cancer cases and 15% of all cancer deaths [1]. Local therapy for nonmetastatic breast cancer consists of surgical resection through mastectomy or breast-conserving surgery (in combination with radiation therapy), and sampling or removal of axillary lymph nodes [2,3]. Depending on tumor histology, grade, stage and molecular subtype, patients also receive systemic therapy that is given preoperatively, postoperatively or both [4,5]. The advantage of neoadjuvant systemic therapy (NAT) is that the extent and morbidity of surgery can be reduced, and the finding of residual disease can guide recommendations related to adjuvant therapy, improving patient outcomes [6,7,8,9,10].

Approximately one third of all breast cancer patients that receive NAT achieve a pathological complete response (pCR), defined as no residual vital invasive tumor cells in the resected tissue [11]. In these patients, it can be questioned as to whether any surgical resection was needed [12,13,14]. However, a major challenge in pursuing a surgery-free treatment strategy for patients that have achieved a pCR is the preoperative identification of a pCR. Current imaging modalities, such as ultrasound, magnetic resonance imaging (MRI) and [^18^F]2-fluoro2-deoxy-D-glucose (FDG) positron emission tomography (PET)/computed tomography (CT), or minimally invasive biopsies are not sufficiently accurate to identify a pCR before surgery [15,16,17,18,19].

In human epidermal growth factor receptor 2 (HER2)-positive breast cancer patients, the pCR rate after NAT is even higher than in the other subtypes, up to 68% [20,21,22,23]. HER2 is a transmembrane receptor tyrosine kinase that is involved in the regulation of cell proliferation, survival, differentiation and angiogenesis [24,25]. Approximately 20% of invasive ductal breast malignancies are classified as HER2-positive as a result of *ERBB2* gene amplification or the subsequent overexpression of the HER2 protein [26,27]. Most HER2-positive breast cancer patients receive NAT that includes trastuzumab and pertuzumab, monoclonal antibodies targeting the extracellular and dimerization domain of HER2, respectively, a well-established therapeutic strategy that has positively affected prognosis [27,28,29].

As a result of this effective NAT, HER2-positive tumors are associated with the highest likelihood of a complete response. However, their NAT response evaluation seems particularly difficult compared to other breast cancer subtypes, possibly due to a reduced contrast uptake as a result of impaired angiogenesis of the tumor caused by HER2-targeted therapies [15,19,30,31,32,33,34]. A radiologic complete response (rCR) on MRI after NAT for HER2-positive breast cancer corresponds to a pCR in only 42–73% of patients [19,22]. This variation can partly be explained by differences in hormonal receptor status of HER2-positive tumors. The MRI false negative rate is especially high in estrogen receptor (ER)- and progesterone receptor (PR)-positive tumors, due to the lower pCR rate of these tumors [22,35]. Therefore, enhanced preoperative diagnostic techniques are warranted for accurate identification of HER2-positive breast cancer patients with a pCR after NAT, to pave the way for a surgery-free treatment strategy.

A diagnostic technique with the potential to improve the preoperative identification of HER2-positive breast cancer patients with a pCR is HER2-targeted PET/CT-imaging using Zirconium-89 (^89^Zr)-radiolabeled trastuzumab. Although loss of HER2 expression after NAT has to be acknowledged as a potential disadvantage, this tumor-targeted technique specifically highlights residual tumor cells and could therefore have an improved diagnostic accuracy after NAT compared to conventional imaging [36]. In a first-in-human feasibility study, fourteen patients with HER2-positive metastatic breast cancer received 37 MBq ^89^Zr-trastuzumab (~1.5 mg), replenished to either 10 or 50 mg with nonradioactive trastuzumab, intravenously [37]. The 50 mg total mass dose in trastuzumab-naïve patients and the 10 mg total mass dose in patients already on trastuzumab treatment were adequate for PET imaging of HER2-positive metastases. The optimal time for assessing uptake of ^89^Zr-trastuzumab in tumor lesions was found to be 4–5 days after the injection [37]. A subsequent study, which investigated radiation dosimetry, safety and optimal imaging parameters, confirmed the optimal imaging time of 5 ± 1 day post-injection for visualizing HER2-positive primary breast tumors and metastases with the highest image quality and tumor-to-nontumor contrast [38]. To date, research has mainly focused on using ^89^Zr-trastuzumab PET/CT to detect breast cancer metastases and non-invasively determine HER2 status [39,40,41,42]. Although these studies have demonstrated a strong correlation between the ^89^Zr-trastuzumab PET/CT signal and the presence of HER2-positive breast cancer cells, the potential role of ^89^Zr-trastuzumab PET/CT in NAT response evaluation has not yet been investigated. Currently, there is no sufficiently adequate non-invasive diagnostic modality to assess response after NAT in HER2-positive breast cancer patients. The aim of this clinical feasibility trial was to assess whether ^89^Zr-trastuzumab PET/CT can be used for accurate, non-invasive NAT response evaluation and pCR identification. To this end, HER2-positive breast cancer patients scheduled to undergo NAT and subsequent surgery underwent a ^89^Zr-trastuzumab PET/CT scan both before and after NAT. These PET/CT images were correlated with standard-of-care MR images and histopathology of both primary tumor and lymph nodes.

## 2. Patients and Methods

### 2.1. Patient Population

This prospective, single arm, open label pilot study was approved by the medical ethics committee of the Leiden University Medical Center (LUMC) in the Netherlands. All patients provided written informed consent before any study-related procedure was performed.

Female patients aged 18 years or older with HER2-positive breast cancer scheduled to undergo NAT and subsequent surgery at the Haaglanden Medical Center (HMC) or the LUMC were eligible. HER2-positivity was defined as an immunohistochemistry score (IHC) of 3+ or an IHC score of 2+ followed by in situ hybridization showing a HER2/CEP17 ratio ≥ 2.0, according to the American Society of Clinical Oncology guidelines [43]. Other eligibility criteria included a tumor size ≥ 5 mm diameter according to anatomical imaging data and a World Health Organization performance status of 0–2. Patients with a history of hypersensitivity to immunoglobulins or clinically significant cardiovascular, pulmonary, renal or liver disease, prior radiotherapy to or a prosthesis in the target breast and pregnant or lactating women, as well as patients with any inabilities not allowing compliance with the study procedures, were excluded.

Surgery consisted of lumpectomy or mastectomy, combined with a sentinel lymph node biopsy (SNB) in patients with no evidence of lymph node involvement prior to neoadjuvant therapy (cN0), and a MARI procedure (i.e., selective removal of a tumor-positive lymph node that was marked with radioactive iodine) in patients with confirmed lymph node involvement (cN+) [44,45].

### 2.2. ^89^Zr-Trastuzumab PET/CT

Patients with HER2-positive breast cancer scheduled to undergo NAT and subsequent surgery underwent two ^89^Zr-trastuzumab-PET/CT scans (Vereos, Philips Heathcare, Best, The Netherlands) in prone position, one pre-NAT (PET/CT-1) and one post-NAT (PET/CT-2), at the LUMC. Clinical grade ^89^Zr-trastuzumab was produced at the Amsterdam University Medical Center under cGMP conditions. For both the pre-NAT and post-NAT PET/CT scan, patients received 37 MBq (±10%) ^89^Zr-trastuzumab (~1.5 mg), replenished to 50 mg with nonradioactive trastuzumab, intravenously, based on previous phase I studies [37,38]. All subjects were monitored for adverse reactions (e.g., dyspnea, chest tightness, palpitations, fever, rigors or hypotension) during administration of ^89^Zr-trastuzumab. Four days post-injection, the shoulders to mid-thorax (diaphragm) was scanned in two bed positions with 5 min/bed position in combination with a low dose CT scan (120 kVp, 35 mAs, 5-mm slice thickness) for attenuation correction and anatomic reference. For the qualitative assessment, PET/CT scans were reconstructed using a time-of-flight blob-based 3D iterative reconstruction algorithm (blobTOF; 3 iterations and 9 subsets) followed by a 3 mm full-width at half maximum (FWHM) post-reconstruction Gaussian filter, with an image matrix of 2 × 2 × 2 mm^3^ (non-EARL PET images). For the quantitative assessment, PET/CT scans were reconstructed in compliance with the EARL Zr89 accreditation program using blobTOF (3 iterations and 15 subsets) followed by a 5.5 mm FWHM post-reconstruction Gaussian filter, with an image matrix of 4 × 4 × 4 mm^3^ (EARL PET images) [46]. After reconstruction, all PET images were converted to standardized uptake values (SUV) by normalizing the measured radioactivity concentrations (kBq·mL^−1^) to the injected dose (MBq) and the patient’s body weight (kg).

### 2.3. Image Analysis

Standard-of-care MR images in prone position before (MRI-1) and after NAT (MRI-2) were re-assessed specifically for this study by two dedicated breast radiologists (M.W. and E.V.) who were blinded for pathology results. Images were evaluated for tumor size, suspicious lymph nodes, speed and intensity of (pathological) contrast enhancement, washout and diffusion restriction. On MRI-2, response evaluation was performed according to the RECIST1.1 guidelines [47]. An rCR of the primary tumor was defined as the absence of invasive or pathologic contrast enhancement in the original tumor regions. A nodal rCR was defined as the absence of suspicious lymph nodes on MRI-2.

PET images were interpreted and analyzed by two dedicated nuclear medicine physicians (L.P. and L.G.) who were blinded for pathology results. PET/CT-1 images were correlated with MRI-1 images to confirm that tumor location on both imaging modalities was identical. PET/CT-2 images were interpreted without consulting the MRI-2 images.

The ^89^Zr-trastuzumab-PET/CT images were analyzed using the IntelliSpace Portal Software version 12.1 (Philips Healthcare, Best, The Netherlands). First, the non-EARL PET/CT scans were qualitatively assessed for ^89^Zr-trastuzumab uptake in the primary tumor and lymph nodes, tumor demarcation and uptake pattern. On PET/CT-2, an rCR was defined as visual disappearance of ^89^Zr-trastuzumab uptake in the tumor region.

Subsequently, quantitative analysis was performed on the EARL reconstructions. For fixed mass doses of ^89^Zr-labeled antibodies, the SUV and tumor-to-blood ratio (TBR) are validated measures for quantification of irreversible tracer uptake in tumors [48]. The TBR is defined as SUV in the tumor divided by the SUV in blood (plasma), obtained from sampling [49]. The assessment of the radioactivity within a blood pool region (also known as the image-derived input function) can be used as an alternative to blood sampling [48,50].

^89^Zr-trastuzumab uptake was quantified as mean and/or maximum SUV in manually drawn volumes of interest around the primary tumor, involved lymph nodes and healthy tissue (contralateral breast, liver and descending aorta), using semi-automatic tumor segmentation, based on subject individual thresholding. The value of the threshold was based on consensus between the two nuclear medicine physicians.

Using the SUV values of the manually drawn volumes of interest, the standardized uptake value ratio (SUV_R_), defined as SUV_max_ of the BC lesion divided by SUV_mean_ of healthy tissue in the contralateral breast, and the tumor-to-blood ratio (TBR), defined as SUV_max_ of the BC lesion divided by SUV_mean_ of the blood pool of the descending aorta, were calculated for both PET/CT scans for each patient [48]. Subsequently, the ∆SUV_R_%, defined as the percentual difference between the SUV_R_ on PET/CT-1 and SUV_R_ on PET/CT-2, and ∆TBR%, defined as the percentual difference between the TBR on PET/CT-1 and TBR on PET/CT-2, were calculated. Of the lymph nodes that showed ^89^Zr-trastuzumab uptake on PET/CT-1 and/or PET/CT-2, the tumor volume (TV) and SUV_max_ were calculated.

### 2.4. Histopathological Examination

All diagnostic biopsies and resection specimens were evaluated at the LUMC by a board-certified dedicated breast pathologist (D.C.). Routine assessment of tumor status following hematoxylin and eosin (H&E) staining and additional HER2 staining was performed on all resected lesions. Primary tumor pCR was defined as the absence of invasive tumor cells in the resected specimen, irrespective of remaining in situ lesions (ypT0/ypTis). Nodal pCR was defined as the absence of tumor cells or isolated tumor cells (≤0.2 mm or less than 200 cells). Residual nodal disease was defined as the presence of micrometastases (>0.2 and ≤2.0 mm) and/or macrometastases (>2.0 mm). For the quantification of residual disease, the Residual Cancer Burden (RCB) score was used [51].

### 2.5. Statistical Analysis

Statistical analyses were performed using SPSS (version 25.0; IBM Corporation, Armonk, NY, USA). Demographic and clinical characteristics were summarized by descriptive statistics. Descriptive statistics are depicted as mean (SD) or median (IQR). For both ^89^Zr-trastuzumab PET/CT and MRI, the accuracy for qualitative response assessment was calculated. To investigate the difference in ∆SUV_R_ (%) of the primary tumor (area) between patients with a pCR and patients with a pPR, a Mann–Whitney U test was performed. The same test was used to assess the difference in ∆TBR (%) between patients with a pPR and pCR. For all analyses, the detection of residual disease via imaging or pathology analysis was considered as positive and pCR via imaging or pathology analysis was considered as negative. A two-sided *p*-value of <0.05 was considered to be statistically significant.

## 3. Results

### 3.1. Patient Characteristics

Between June 2019 and March 2022, six HER2-positive breast cancer patients were enrolled in the trial. PET/CT-1 and PET/CT-2 were performed in all six patients. Patient and tumor characteristics are summarized in Table 1. Median age at diagnosis was 51 years (IQR 47–56.25). All six patients had a HER2-positive invasive carcinoma, no special type (NST) and received six or nine three-week cycle courses of paclitaxel, trastuzumab, carboplatin and pertuzumab (PTC-PTZ). Five patients underwent lumpectomy with the SNB or MARI procedure. One patient underwent mastectomy with SNB. One tumor was ER-positive. One tumor was ER- and PR-positive. The median Ki-67 was 37.5% (IQR 25–60). Four out of six patients (66.7%) had a pCR of the primary tumor, of which two had remaining in situ disease (Tis). Two out of six patients (33.3%) had a pathological partial response (pPR) of the primary tumor after NAT. Three patients had one or more tumor-positive lymph nodes at diagnosis. These patients all achieved a nodal pCR after NAT. The median time interval between MRI-1 and MRI-2, and between PET/CT-1 and PET/CT-2 was 147 (IQR 74.5–164.25) and 139 (IQR 130–165.5) days, respectively. The median time interval between MRI-1 and PET/CT-1, and MRI-2 and PET/CT-2 was 26 (IQR 18.75–31.25) and 48 (IQR 9.25–77.5) days, respectively. The amount of time that passed between MRI-1 and PET/CT-1 can be explained by the logistics with regard to acquiring patient informed consent at the outpatient clinic and subsequent ^89^Zr-trastuzumab production and delivery, which required at least one week. The MRI-2–PET/CT-2 time interval was caused by the same logistical challenges relating to ^89^Zr-trastuzumab and the fact that in three patients (2, 3, and 6) MRI-2 was performed before the official last day of NAT. The median time interval between the last day of NAT and PET/CT-2 was 14 days (IQR 6–20.5). PET/CT-1 detected all primary tumors, with a median SUV_max_, SUV_R_ and TBR of 5.55, 16.65 and 1.52, respectively.

### 3.2. Treatment Response Evaluation of the Primary Tumor

At the final histopathological examination of the primary tumor resection, four patients had achieved a pCR and two a pPR after NAT. The post-NAT response assessment using standard-of-care MRI and the ^89^Zr-trastuzumab PET/CT are summarized in Table 2. Qualitative assessment of the (non-EARL) ^89^Zr-trastuzumab PET/CT and MRI yielded an accuracy of 66.7% (4/6 correct) and 83.3% (5/6 correct), respectively. Negative predictive values of PET/CT and MRI were 66.7% and 80%, respectively. PET/CT-2 had two false negative readings (complete instead of partial response) and MRI-2 had one false negative reading (complete instead of partial response). This means that using qualitative (visual) assessment alone, the ^89^Zr-trastuzumab PET/CT missed residual tumor in two of our patients. Both patients had a residual cancer burden (RCB)-class of II (RCB score 1.37–3.28).

Subsequent quantification of the (EARL) PET/CT signal showed a median ∆SUV_R_% of −48% (range 0.18) and −90% (IQR −85%–−93%) for a pPR and pCR (*p* = 0.133), respectively.

In contrast, the median ∆TBR% did not differ substantially between patients with a pPR (−79%, range 16.26) and pCR (−94%, IQR −92%–−95%) (*p* = 0.133).

**Figure 1:**
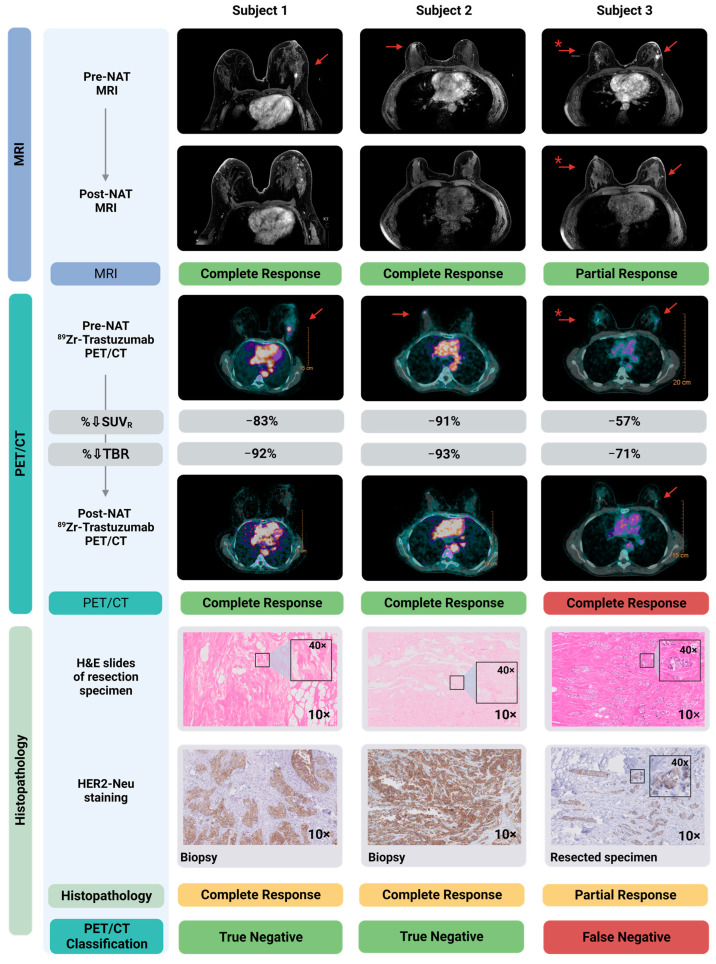
Diagnostic MRI- and ^89^Zr-trastuzumab-PET/CT images pre- and post-NAT correlated to histopathology in the resected primary tumor. Shown here are three subjects, two with a pCR and one with a pPR. MRI-1 and PET/CT-1 localized all breast cancer lesions accurately. MRI-2 accurately specified these three patients for complete/partial response. Qualitative assessment of PET/CT-2 accurately identified the two patients with a pCR. As shown in the right panel, qualitative assessment of PET/CT-2 classified the subject with a pPR incorrectly as a pCR, and therefore is a false negative. Of note, the SUV_R_% in this patient (−57%) is considerably lower compared to the two patients with a pCR (−91% and −83%). Shown in the lower panels are the hematoxylin and eosin (H&E) staining of the resected specimen, as well as HER2 staining on, respectively, the diagnostic biopsy (Subject 1 and 2) and the resected primary tumor specimen containing residual tumor (Subject 3). * Patient with bilateral lesion; left: invasive carcinoma; right: ductal carcinoma in situ (DCIS). Created with Biorender.com. Abbreviations: H&E, hematoxylin and eosin staining; MRI, magnetic resonance imaging; NAT, neoadjuvant systemic therapy; pCR, pathological complete response; pPR, pathological partial response; SUV_R_, standardized uptake value ratio; TBR, tumor-to-blood ratio.

Figure 1 shows diagnostic MRI and ^89^Zr-trastuzumab PET/CT images of the primary tumor, pre- and post-NAT, from three representative patients, correlated to definitive histopathology in the resected specimen. The PET/CT-1 scan accurately identified the primary tumor in all three patients. Two of these illustrated patients had achieved a pCR that was accurately diagnosed by both MRI-2 and PET/CT-2. One patient had residual disease that was missed by qualitative assessment of PET/CT-2.

### 3.3. Treatment Response Evaluation of Metastatic Lymph Nodes

Three out of six patients had biopsy-proven tumor-positive lymph nodes at the time of diagnosis. After NAT, none of these three patients had residual vital tumor cells in the resected lymph node(s), i.e., all had a nodal pCR.

Qualitative assessment of MRI-1 and PET/CT-1 resulted in an accuracy for the detection of metastatic lymph nodes of 50% and 100%, respectively (Table 3). In two patients, MRI-1 incorrectly classified tumor-negative lymph nodes as suspect (false positive). In one patient, biopsy-proven tumor-positive lymph nodes were classified as unsuspected on MRI-1 (false negative). The PET/CT-1 correctly identified the metastatic lymph nodes in all three patients.

Of the two patients with metastatic lymph nodes that were accurately diagnosed by MRI-1, one was correctly labeled as rCR on MRI-2. The other patient was labeled as partial response but was in fact a pCR (false positive), corresponding to an accuracy of 50%. PET/CT-2 correctly identified two of the three patients with a nodal pCR, corresponding to an accuracy of 66.7%. The third patient with a nodal pCR was classified as a partial response by PET/CT-2 (and was therefore a false positive). In this patient, quantification of the PET/CT signal showed a ∆SUV_max_%, the percentual difference between PET/CT-1 and PET/CT-2, of −88%. Because no lymph nodes with a confirmed pPR were imaged, this difference could not be compared to a ∆SUV_max_% in a lymph node with residual tumor.

**Figure 2:**
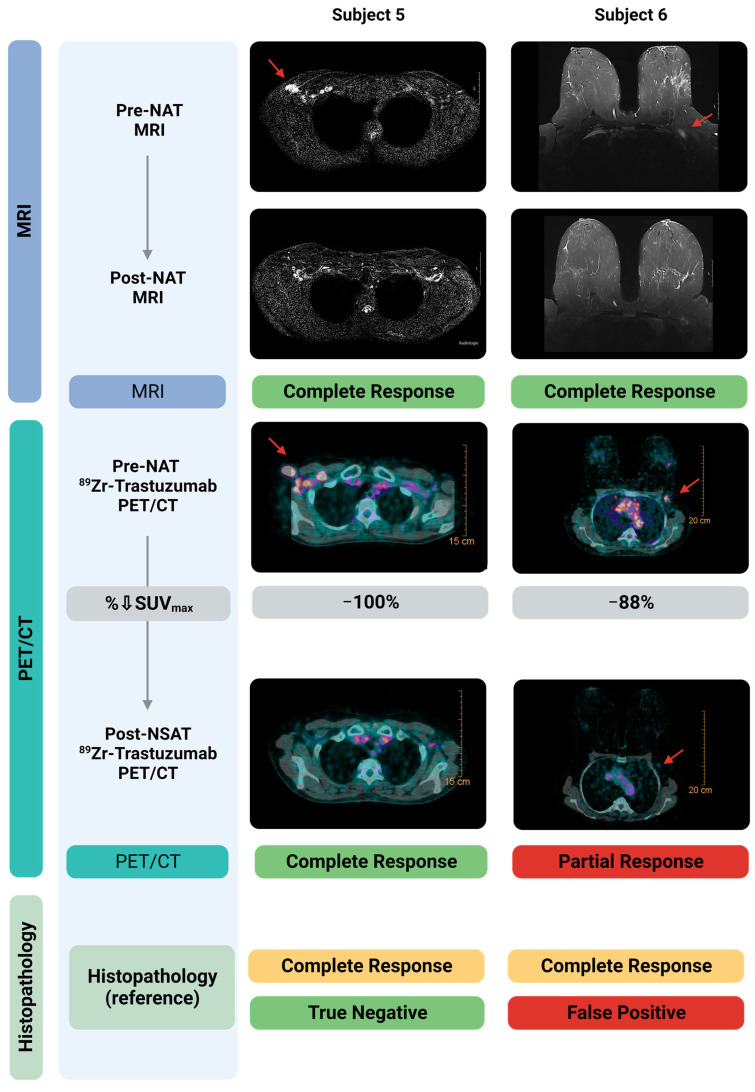
Diagnostic MRI- and ^89^Zr-trastuzumab-PET/CT images pre- and post-NAT correlated to histopathology in the resected sentinel lymph node. Shown here are two subjects, both with a nodal pCR. Pre-NAT MRI and ^89^Zr-trastuzumab-PET/CT localized the tumor positive lymph nodes accurately. Post-NAT MRI accurately specified both patients for complete response. Post-NAT ^89^Zr-trastuzumab-PET/CT accurately specified one of the two patients with a pCR. As shown in the right panel, ^89^Zr-trastuzumab-PET/CT classified one subject with a pCR incorrectly as a pPR, and therefore is classified as False Positive. Created with Biorender.com. Abbreviations: H&E, hematoxylin and eosin staining; MRI, Magnetic Resonance Imaging; NAT, neoadjuvant systemic therapy; pCR, pathological complete response; pPR, pathological partial response; SUV_max_, Maximum Standardized Uptake Value.

Figure 2 shows representative diagnostic MRI and ^89^Zr-trastuzumab PET/CT images, pre- and post-NAT, from two representative patients, correlated to histopathology in the resected lymph node. The MRI-1 and PET/CT-1 scan accurately identified the metastatic lymph nodes. Both patients had achieved a nodal pCR that was accurately diagnosed on MRI-2. In one patient, the PET/CT-2 misidentified one of these patients as a partial instead of complete response (false positive).

### 3.4. Treatment Response Evaluation of Ductal Carcinoma In Situ (DCIS)

One patient (Subject 3) presented with bilateral breast cancer. The tumor in the left breast was diagnosed as invasive breast cancer NST, and the right breast tumor as ductal carcinoma in situ (DCIS) (Figure 1). According to national and international guidelines, because patients with residual in situ disease after NAT are classified as a pCR, the DCIS lesion in the right breast was not included in the accuracy analysis described in Section 3.2. However, qualitative and quantitative response assessment of the DCIS lesion was performed (Table A1). 

The DCIS lesion had a pPR after NAT. This response was diagnosed correctly by MRI-2. Qualitative assessment of PET/CT-2 classified the response as complete instead of partial (false negative). Quantifying the ^89^Zr-trastuzumab uptake showed a ΔSUV_R_ of −64%, which is considerably (although not significantly, *p* = 0.2) lower than the median ∆SUV_R_ of −90% in patients with a pCR. However, the two patients (Subject 1 and 4) with a pCR that had residual DCIS had a ΔSUV_R_ of respectively −83% and −94%.

## 4. Discussion

The aim of this feasibility study was to investigate if ^89^Zr-trastuzumab PET/CT imaging can be used for accurate response evaluation of the primary tumor and lymph nodes after NAT in patients with HER2-positive breast cancer. To this end, patients received ^89^Zr-trastuzumab PET/CT both before and after NAT. In line with previous studies, we found that pre-NAT ^89^Zr-trastuzumab PET/CT allows the detection of HER2-positive breast cancer lesions, with a median SUV_max_, SUV_R_ and TBR of 5.55, 16.65 and 1.52, respectively [39]. We have demonstrated that qualitative assessment of ^89^Zr-trastuzumab PET/CT for treatment response evaluation of the primary tumor has a moderate accuracy and negative predictive value of 66.7%. However, quantitative assessment of the PET/CT images shows a considerable (although, in this limited patient series, not statistically significant) difference in the percentual decrease of the SUV_R_ between patients with a pPR and pCR of the primary tumor (−48% vs. −90%, *p* = 0.133). This difference suggests that ^89^Zr-trastuzumab PET/CT could be used to accurately distinguish between patients with and without residual invasive breast cancer based on the ∆SUV_R_%. In contrast, the percentual decrease of the TBR, calculated using an image-derived input function, did not differ substantially between patients with a pPR and pCR (−79% vs. −94%, *p* = 0.133) and therefore might be less suitable for response evaluation. Additionally, we show that qualitative assessment of the pre- and post-NAT PET/CT, respectively, had a 100% and 66.7% accuracy for the detection of metastatic lymph nodes. However, the number of metastatic lymph nodes that were imaged was limited.

To the best of our knowledge, the current study is the first that investigated ^89^Zr-trastuzumab PET/CT for treatment response evaluation in breast cancer. Qualitative assessment of the ^89^Zr-trastuzumab PET/CT images in our patients resulted in a diagnostic performance comparable with that of MRI and/or [^18^F]FDG-PET/CT described in larger breast cancer response evaluation studies [19,22,52,53]. However, for a safe surgery-free treatment strategy in patients with an rCR, a higher diagnostic accuracy, especially negative predictive value, is imperative. Our results suggest that quantitative assessment could enhance the diagnostic performance of ^89^Zr-trastuzumab PET/CT by using the percentual SUV_R_ decrease difference between patients with and without residual disease. The SUV has previously been validated for the quantification of irreversible tracer uptake in tumors for fixed mass doses of ^89^Zr-labeled antibodies, which is the case in this study [48]. Moreover, it has previously been shown that a relative change in SUV can be used to differentiate between patients with and without a pCR after NAT using [^18^F]FDG-PET/CT [54,55].

In our study, qualitative assessment of ^89^Zr-trastuzumab PET/CT wrongly qualified two patients as a complete responder. Both tumors were ER-positive, and one was also PR-positive. The residual tumor cells found in the resected tissue of these patients were still HER2-positive. An explanation for the false negative PET/CT readings could be that the ^89^Zr-trastuzumab total dose of 50 mg that was administered for the post-NAT was too high. Due to downregulation of HER2 caused by trastuzumab and pertuzumab treatment, and the fact that a very small volume of residual tumor cells remain after NAT, the number of available binding sites for ^89^Zr-trastuzumab significantly decreases. If the same ^89^Zr-trastuzumab total dose is administered, this could result in saturation of HER2 with unlabeled (i.e., cold) trastuzumab and, consequently, attenuation of the PET/CT signal [37]. Possible solutions could be to administer a lower total ^89^Zr-trastuzumab dose for the post-NAT scan or to use a HER2-targeted radiotracer that does not compete with trastuzumab or pertuzumab, which are currently being developed [37,56]. Additionally, in one patient with a nodal pCR, qualitative assessment of the post-NAT ^89^Zr-trastuzumab PET/CT incorrectly labeled the nodal response as partial. It could be that the lymph node was reactive, leading to Fc receptor-mediated ^89^Zr-trastuzumab accumulation.

The vital tumor rest and corresponding RCB scores in the two patients with a pPR were 35% and <10%, and 1.6 and 1.8, respectively (both class II). The remaining ^89^Zr-trastuzumab PET/CT signal caused by these scattered residual tumor cells could evidently not be detected with qualitative assessment after thresholding. Interestingly, the patient with the lower vital tumor rest and RCB score also had a lower ∆SUV_R_ (−39% vs. −57%), indicating that this quantitative variable accurately reflects the amount of residual tumor.

Although our results are promising, the current study has some limitations. First, the sample size in this feasibility study was small and therefore did not permit the generation of reliable receiver-operating characteristic (ROC) curves to determine cut-off values with optimal sensitivity and specificity for the quantitative ^89^Zr-trastuzumab PET/CT variables [57]. These cut-off values have to be determined and validated in a larger patient cohort. In that same cohort, the diagnostic accuracy of ^89^Zr-trastuzumab PET/CT in hormonal receptor-positive and -negative HER2-positive breast tumors should also be investigated. Secondly, in line with national and international guidelines, we included DCIS as a primary tumor pCR. However, it remains unclear if a surgery-free treatment strategy could be applied in patients with a pCR of the invasive tumor but with residual DCIS.

## 5. Conclusions

In conclusion, NAT response evaluation in HER2-positive breast cancer patients using ^89^Zr-trastuzumab PET/CT imaging is feasible. Especially quantitative assessment of the ^89^Zr-trastuzumab PET/CT images shows the potential to accurately identify patients with a pCR of the invasive component of the primary tumor, although procedures for HER2-targeted PET/CT might need further optimization. Validation in a larger patient cohort has to demonstrate if a sufficiently high diagnostic accuracy can be achieved to allow for a safe surgery-free treatment strategy in HER2-positive breast cancer.

## Figures and Tables

**Table 1:** Demographic and clinical characteristics.

Subject Number	Age at Diagnosis (Years)	Receptor Status Biopsy	Tumor Grade	Ki-67	cTNM Stage	Type of NAT	Type of Surgery	Time between PET/CT-1 and PET/CT-2 (Days)	ypTNM	HER2 Status Resection Specimen
**1**	48	ER-/PR-/HER2+	2	35%	cT1cN0M0	6 courses PTC-PTZ	Lumpectomy + SNB	132	ypTisN0M0	NA
**2**	57	ER+/PR-/HER2+	3	30%	cT2N0M0	6 courses PTC-PTZ	Lumpectomy + SNB	134	ypT0N0M0	NA
**3**	48	ER+/PR+/HER2+	2	10%	cT1cN0M0	6 courses PTC-PTZ	Mastectomy + SNB	118	ypT1cN0M0	HER2+
**4**	54	ER-/PR-/HER2+	3	60%	cT2N1M0	6 courses PTC-PTZ	Lumpectomy + MARI	144	ypTisN0M0	NA
**5**	44	ER-/PR-/HER2+	3	60%	cT2N2M0	6 courses PTC-PTZ	Lumpectomy + MARI	153	ypT0N0M0	NA
**6**	56	ER+/PR-/HER2+	3	40%	cT2N2M0	9 courses PTC-PTZ	Lumpectomy + MARI	203	ypT1aN0	HER2+

Notes: Depicted are the demographics and clinical data of the patients enrolled in the study. Abbreviations: MARI, marking the axilla with radioactive iodine seeds; NA, not applicable; NAT, neoadjuvant systemic therapy; ER, estrogen receptor; PR, progesterone receptor; PTC-PTZ, paclitaxel, trastuzumab, carboplatin + pertuzumab; SNB, sentinel lymph node biopsy.

**Table 2:** Response assessment and concordance of ^89^Zr-trastuzumab uptake values and RCB score of the primary tumor.

Subject Number	Dimension Lesion Based on MRI-1 (mm)	Radiologic Response MRI-2	Radiologic Response PET/CT-2	Pathological Response (Reference)	Vital Tumor Rest in Resection Specimen (%)	Largest (Rest) Invasive Lesion Diameter Based on Pathology (mm)	TV (mm^3^) PET/CT-1	TV (mm^3^) PET/CT-2	∆TV %	SUV_max_ PET/CT-1–PET/CT-2	SUV_R_ PET/CT-1–PET/CT-2	∆SUV_R_ %	TBR PET/CT-1–PET/CT-2	∆TBR%	RCB- Score	RCB-Class
**1**	19 × 9	Complete (TN)	Complete (TN)	Complete	0	-	6.3	0.0	−100%	6.7–0.78	22.3–3.7	−83%	1.46–0.12	−92%	NA	0
**2**	12 × 8	Complete (TN)	Complete (TN)	Complete	0	-	1.5	0.0	−100%	4.4–0.40	11.0–1.0	−91%	1.19–0.08	−93%	NA	0
**3**	18 × 14	Partial (TP)	Complete (FN)	Partial	35%	18	1.8	0.0	−100%	1.4–0.60	7.0–3.0	−57%	1.0–0.29	−71%	1.8	II
**4**	21 × 15	Complete (TN)	Complete (TN)	Complete	0	-	9.5	0.0	−100%	11.0–0.50	27.5–1.7	−94%	2.68–0.12	−96%	NA	0
**5**	47 × 19	Complete (TN)	Complete (TN)	Complete	0	-	21.6	0.8 *	−96%	19.1–1.1	95.5–11.0	−88%	5.03–0.27	−95%	NA	0
**6**	47 × 33	Complete (FN)	Complete (FN)	Partial	<10%	5	19.3	0.0	−100%	3.46–0.30	8.2–5.0	−39%	1.57–0.20	−87%	1.6	II

Notes: Assessment of response after NAT as depicted based on MRI and ^89^Zr-trastuzumab-PET/CT, referenced to pathological response in the resected primary tumor. In subject 3 and 6 a discrepancy between ^89^Zr-trastuzumab-PET/CT and pathology was found. These subjects had a pPR, with a vital invasive tumor rest, and are therefore classified as False Negative (FN). Subjects 1, 2, 4 and 5 had a pCR by imaging and pathology, classified as True Negative (TN). ∆TV, ∆SUV_R_ and ∆TBR changes are calculated as the difference (decrease) between PET/CT-1 and PET/CT-2; the outcome is expressed as %. * Artifact of in situ localizing iodine-125 seed of initial BC lesion. Abbreviations: NAT, neoadjuvant systemic therapy; FN, False Negative; MRI, Magnetic Resonance Imaging; NA, not applicable; pCR, pathological complete response; pPR, pathological partial response; SUV, Standardized Uptake Value; TBR, tumor-to-blood ratio; TV, tumor volume; TP, True Positive; TN, True Negative.

**Table 3:** Response assessment and concordance ^89^Zr-trastuzumab uptake values of the lymph nodes.

Subject Number	Biopsy-Proven Tumor-Positive Lymph Node(s) pre-NAT	Suspect Lymph Nodes on pre-NAT US	Suspect Lymph Nodes on MRI-1	Location Suspect Lymph Nodes on MRI-1	Suspect Lymph Node Dimensions on MRI-1 (in mm)	Radiologic Nodal Response MRI-2	^89^Zr-Trastuzumab Uptake in Lymph Nodes PET/CT-1	Location Suspect Lymph Nodes on PET/CT-1	TV (mm^3^) Lymph Nodes on PET/CT-1	SUV_max_ Lymph Nodes on PET/CT-1	∆SUVmax %	Radiologic Nodal Respons PET/CT-2	Number of Resected Lymph Nodes	Vital Tumor in Resected Lymph Node(s)
**1**	No	No	Yes (FP)	1. Axilla	1. 13 × 5	Complete *	No (TN)	NA	NA	NA	NA	NA	1	No
**2**	No	Yes	Yes (FP)	1. Axilla	1. 9 × 6	Complete *	No (TN)	NA	NA	NA	NA	NA	1	No
**3**	No	No	No (TN)	NA	NA	NA	No (TN)	NA	NA	NA	NA	NA	1	No
**4**	Yes	Yes	No (FN)	NA	NA	NA	Yes (TP)	1. Axilla 2. Axilla	1. 2.6 2. 1.5	1. 12.3 2. 4.1	1. −100% 2. −100%	Complete (TN)	1	No
**5**	Yes	Yes	Yes (TP)	1. Axilla 2. Axilla 3. Axilla	1. 20 × 13 2. 10 × 8 3. 13 × 7	Complete (TN)	Yes (TP)	1. Axilla 2. Axilla 3. Axilla 4. Parasternal	1. 5.5 2. 11.0 3. 2.0 4. 0.2	1. 15.9 2. 12.6 3. 15.4 4. 3.2	1. −100% 2. −100% 3. −100% 4. −100%	Complete (TN)	8 **	No
**6**	Yes	Yes	Yes (TP)	1. Axilla 2. Axilla 3. Axilla 4. Axilla	1. 13 × 26 2. 6 × 12 3. 5 × 11 4. 12 × 17	Partial (FP)	Yes (TP)	1. Axilla 2. Axilla	1. 5.95 2. 2.49	1. 4.46 2. 2.22	1. −88% 2. −100%	Partial (FP)	1	No

Notes: Assessment of nodal response after NAT based on MRI and ^89^Zr-trastuzumab-PET/CT, referenced to pathological response in the resected sentinel lymph node. In subject 6 a discrepancy between ^89^Zr-trastuzumab-PET/CT and pathology was found, therefore classified as False Positive. This subject had a nodal pCR, with no vital tumor rest. ∆SUV_max_ changes are calculated as the difference (decrease) between PET/CT-1 and PET/CT-2, and the outcome is expressed as %. * MRI-1 was False Positive (the radiologists were blinded for pathology). Therefore MRI-2 could be classified as rCR when in fact there was no biopsy-proven tumor present before NAT. ** In addition to the MARI procedure lymph node, additional lymph nodes were resected because of intraoperative clinical suspicion. All were tumor-negative. Abbreviations: NAT, neoadjuvant systemic therapy; FP, False Positive; FN, False Negative; MRI, Magnetic Resonance Imaging; NA, Not applicable; SUV, Standardized Uptake Value; TV, Tumor Volume; TP, True Positive; TN, True Negative.

## Data Availability

The data of the current study can be requested via email from the corresponding author.

## Appendix A

**Table A1:** NAT response assessment of DCIS lesion using ^89^Zr-trastuzumab PET/CT.

Subject Number	Dimension Lesion Based on MRI-1 (mm)	Radiologic Response MRI-2	Radiologic Response PET/CT-2	Pathological Response (Reference)	Largest Lesion Diameter Based on MRI-1 (mm)	Vital Tumor Rest in Resection Specimen (%)	Largest (Rest) Lesion Diameter Based on Pathology (mm)	TV (mm^3^) PET/CT-1	TV (mm^3^) PET/CT-2	∆TV %	SUV_R_ PET/CT-1	SUV_R_ PET/CT-2	∆SUV_R_ %	TBR PET/CT-1–PET/CT-2	∆TBR%
**1**	50 × 30	Partial (TP)	Complete (FN)	Partial	50	10	20	11.8	0.0	−100%	7.0	2.5	−64%	1.0–0.24	−76%

Notes: Assessment of response after NAT as depicted based on MRI and ^89^Zr-trastuzumab-PET/CT, referenced to pathological response in the resected DCIS lesion. A discrepancy between ^89^Zr-trastuzumab-PET/CT and pathology was found. This patient had a partial pathological response, with a vital DCIS rest, and is therefore classified as False Negative. ∆TV, ∆SUV_R_ and ∆SUV_max_ changes are calculated as the difference (decrease) between PET/CT-1 and PET/CT-2, the outcome is expressed as %. Abbreviations: NAT, neoadjuvant systemic therapy; FP, False Positive; FN, False Negative; MRI, Magnetic Resonance Imaging; NA, Not applicable; SUV, Standardized Uptake Value; TBR, tumor-to-blood ratio; TV, Tumor Volume; TP, True Positive; TN, True Negative.
